# Tackling Youth Inactivity and Sedentary Behavior in an Entire Latin America City

**DOI:** 10.3389/fped.2018.00298

**Published:** 2018-10-11

**Authors:** Marcio Atalla, Ana Jessica Pinto, Gregore Iven Mielke, Erica Passos Baciuk, Fabiana Braga Benatti, Bruno Gualano

**Affiliations:** ^1^Wellness Center, São Paulo, Brazil; ^2^Clinical Hospital HCFMUSP, School of Medicine FMUSP, University of São Paulo, São Paulo, Brazil; ^3^Postgraduate Program in Epidemiology, Federal University of Pelotas, Pelotas, Brazil; ^4^School of Human Movement and Nutrition Sciences, The University of Queensland, Brisbane, QLD, Australia; ^5^Centro Universitário de Jaguariúna, Jaguariúna, Brazil

**Keywords:** physical activity, sedentarism, healthy lifestyle, school-based intervention, obesity

## Abstract

Real-world interventions are fundamental to bridge the research-practice gap in healthy lifestyle promotion. This study aimed to assess the impact of a 7-month, intensive, city-wide intervention (“Life of Health”) on tackling youth inactivity and sedentary behavior in an entire Latin-American city (Jaguariuna, Brazil). For youth, a program focused on tackling inactivity/sedentary behavior was delivered at every school (*n* = 18). Plausibility assessments (pre-to-post design) were performed with 3,592 youth (out of 8,300 individuals at school age in the city) to test the effectiveness of the intervention. Primary outcomes were physical activity and sedentary behavior. Secondary outcome was BMI *z*-score. Physical activity did not change (0; 95%CI:-2.7–2.8 min/day; *p* = 0.976), although physically inactive sub-group increased physical activity levels (11.2; 95%CI:8.8–13.6 min/day; *p* < 0.001). Weekday television and videogame time decreased, whereas computer time increased. Participants with overweight and obesity decreased BMI *z*-score (-0.08; 95%CI:-0.11−0.05; *p* < 0.001; −0.15; 95%CI:-0.19−0.11; *p* < 0.001). This intervention was not able to change the proportion of physical inactivity and sedentary behavior in youth at a city level. Nonetheless, physically inactive individuals increased PA levels and participants with overweight and obesity experienced a reduction in BMI *z*-score, evidencing the relevance of the intervention. Education-based lifestyle programs should be supplemented with environmental changes to better tackle inactivity/sedentary behavior in the real-world.

## Introduction

Physical inactivity (i.e., the inability to achieve the minimum recommended levels of physical activity [PA]) and sedentary behavior (i.e., any waking behavior in a sitting/laying position with low energy expenditure) are important risks factors associated with childhood obesity and related health problems in most Western countries (1, 2). PA among children and adolescents is believed to be insufficient (3) and low levels of PA seem to continue into adulthood (4). Thus, combating physical inactivity and sedentary behavior during childhood is a key strategy for preventing future health risks (5, 6).

However, a systematic review concluded that a lack of high-quality large-scale studies precluded conclusions on the effectiveness of efforts to promote PA in youth (7). Moreover, community-based interventions tackling sedentary behavior, which has been associated with obesity in youth, independently of physical inactivity (8), are scant.

The “Life of Health” was a real-word, multi-faceted intervention aimed to promote lifestyle changes in an entire Latin-American city with over 50,000 inhabitants. Specifically for youth (approximately 8,300 people), the intervention mainly focused on tackling physical inactivity and sedentary lifestyle concomitantly through a 5-goal program delivered at every city school (*n* = 18). Using plausibility assessments (evaluations that are aimed at making causal statements of effectiveness using observational designs), we aimed to report on the impact of this intervention in the city's youth.

## Methods

The aims, basis and description of all activities/policies pertaining to the “Life of Health” intervention are found in File S1, whereas details about the city where the intervention took place are described in Table S1. Health professionals and researchers, with the financial and operational support of a private company and the local government (i.e., Education and Health Secretaries), jointly designed this program. In the case of youth, the “Life of Health” intervention was focused on increasing PA levels and reducing sedentary behavior. To achieve this, school directors and pedagogy coordinators from every school of Jaguariuna (Sao Paulo, Brazil) were personally trained by PA promotion specialists to disseminate a 5-goal program to all teachers from the city's educational system, which was implemented through group meetings at each school. Teachers were encouraged to accomplish all the goals and adapt them according to their possibilities (e.g., availability of materials and infrastructure). The 5-goal program comprised the following recommendations:

- To reduce, at least, 5 min of sedentary behavior per hourly class, encouraging standing up during roll call, seminars, work groups, etc.
- To spread campaign posters across the school environment, containing positive messages, such as “While chatting, stand up!”; “Sit down only when you're tired!”; “Go to the playground!”; “How about doing sports?”; “Reduce TV time!.”
- To enrich the school environment encouraging physical activity during recreation and/or lunch breaks, providing the students with full access to exercise equipment and devices (e.g., balls, ropes, hula-hoops, etc.).
- To open the school's games fields, sports halls and playground to the local community. [This was particularly relevant in low-income neighborhoods where the school was the only safe environment for engaging in physical activities.]
- To advise parents on the relevance of increasing physical activity and reducing sedentary of their children. In brief, parents were personally counseled to (a) constrain children's time watching TV or playing videogames; (b) advise their children to break up sedentary time during periods of prolonged sitting; (c) spend leisure time with family and/or friends outdoor (e.g., parks, community clubs); (d) register children in sports clubs and/or wellness centers; and (e) provide children with rewards, incentives and/or positive feedback for engaging in physical activities and/or reducing sedentary time.

Throughout the program, members of the intervention team visited every school at least once and maintained weekly telephone contact with the schools' representatives to ensure the implementation of the program, as well as to stimulate teachers to adhere to it.

Primary outcomes were PA level and sedentary behavior, assessed through a validated questionnaire (9). In brief, this tool obtains information about (a) participation in up to 3 types of structured and/or unstructured physical activities and/or sports according to weekly frequency and daily duration, with the sum of the three activities resulting in a final score for PA; and (b) time spent in sedentary activities (i.e., TV watching, playing videogame, and using computer). Because this questionnaire was only validated for children ≥10 years old, it was not applied to participants aged <10 years (*n* = 1,442). Participants were classified as sufficiently physically active if they performed a minimum of 60 min/day of structured and/or unstructured PA during leisure-time (10), and engaging in excessive sedentary time if they spent more than 2 h/day in screen-based behavior (11, 12). Secondary outcome was BMI *z*-score, calculated using the WHO AnthroPlus® software (13).

Children and adolescents from every school were invited to participate in the study through an explanatory document about the intervention sent to parents or legal guardian(s). All individuals who brought the signed informed consent to school prior to assessments were included. Data collection was performed between April and December of 2016. This study was approved by the ethical committee.

Plausibility assessments with a pre-to-post design were performed using generalized estimating equations (GEEs), with the assumption of a linear distribution, id link function, and an exchangeable working correlation for repeated outcomes over time for the participants. The models were adjusted for age, sex, and BMI. Sub-analyses were also conducted accounting for stratus of age [6-12 [or 10-12 for PA/sedentary behavior variables], and 13-17 years], sex, BMI, physical inactivity, and sedentary lifestyle. Data analyses were performed using a statistical package (SPSS, version 17.0). Significance level was set at *p* ≤ 0.05. Data are presented as mean ± SD, absolute numbers, percentages, delta scores, and/or 95%CI.

## Results

This study enrolled 3,592 participants (~43% of all school-aged youth from the city) and 378 were lost to follow up (Figure S1). Participants' demographic characteristics are presented in Table S2.

Overall, PA did not change following the intervention (Pre: 37.2 ± 52.2, Post: 37.4 ± 55.9; difference: 0.2; 95%CI:−2.7 to 2.8 min/day; *p* = 0.976; Table 1). The proportion of physically active participants did not change (−1%; 95%CI: − 9% to 9%; *p* = 0.664; Figure S2). Children decreased PA (Pre: 39.8 ± 54.6, Post: 34.6 ± 53.7; difference: − 5.2; 95%CI: − 9.9 to − 0.4 min/day; *p* = 0.033, Table S3), whereas adolescents tended to increase it (Pre: 35.8 ± 50.8, Post: 39.2 ± 57.2; difference: 3.3; 95%CI: − 0.1 to 6.7 min/day; *p* = 0.057, Table S5). However, physically inactive participants increased PA (Pre: 15.8 ± 17.0, Post: 26.4 ± 43.4; difference: 11.2; 95%CI: 8.8 to 13.6 min/day; *p* < 0.001; Table S11). Sex, BMI, and sedentary lifestyle did not affect the results (Figure S2, Tables S5–S10, S12).

**Table 1:** Physical activity and sedentary behavior before and after the intervention (*n* = 2,036).

**Variable**	**Pre**	**Post**	**Delta change**	**95% CI**	***p***
Physical activity, min/day	37.2 ± 52.2	37.4 ± 55.9	0.0	−2.7 to 2.8	0.976
**SEDENTARY BEHAVIOR, MIN/DAY**					
Television time during weekdays	128.9 ± 105.2	121.7 ± 105.2	−8.5	−12.7 to −4.3	<0.001
Television time during weekend days	79.6 ± 79.4	78.2 ± 83.8	0.0	−3.7 to 3.8	0.980
Time spent playing video games	57.4 ± 101.3	37.8 ± 81.1	−20.9	−25.0 to −16.8	<0.001
Time spent on computer	93.3 ± 139.3	149.0 ± 176.6	63.7	56.5 to 71.0	<0.001

*Data are expressed as mean ± standard deviation, delta change (Post – Pre), and 95% CI of the difference. P-value was adjusted by age, sex, and BMI*.

Overall, television time during weekdays reduced (Pre: 128.9 ± 105.2, Post: 121.7 ± 105.2; difference: −8.5; 95%CI: −12.7 to −4.3 min/day; *p* < 0.001) whereas television time during weekend days did not change (Pre: 79.6 ± 79.4, Post: 78.2 ± 83.8; difference: 0; 95%CI: − 3.7 to 3.8 min/day; *p* = 0.980; Table 1). Time spent playing videogames decreased (Pre: 57.4 ± 101.3, Post: 37.8 ± 81.1; difference: −20.9; 95%CI: −25.0 to −16.8 min/day; *p* < 0.001) while time spent on computer increased (Pre: 93.3 ± 139.3, Post: 149.0 ± 176.6; difference: 63.7; 95%CI: 56.5 to 71.0 min/day; *p* < 0.001; Table 1). The frequency of sedentary participants did not change (2%; 95%CI: −2% to 5%; *p* = 0.097; Figure S3). When participants were sub-divided according to age, sex, BMI, sedentary lifestyle, and physical inactivity, the results followed a similar pattern to the overall sample (Tables S3–S12). The exception was the absence of changes in television time during weekdays for males and underweight participants.

**Figure 1:**
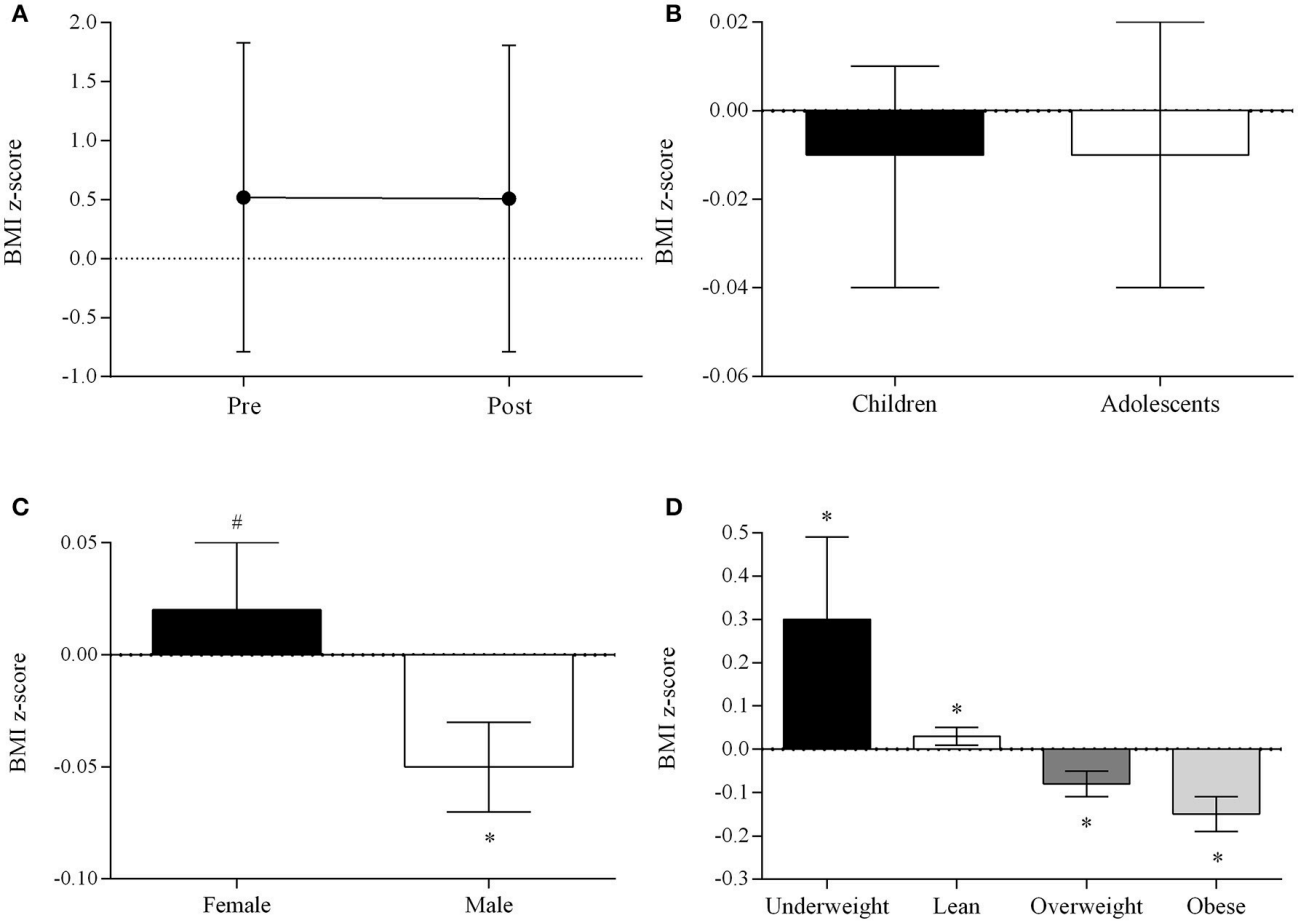
BMI *z*-score before and after the intervention **(A)**, and changes in BMI *z*-score stratified by age **(B)**, sex **(C)**, and BMI **(D)**. Data are expressed as mean ± standard deviation, and delta change (Post - Pre) and 95% CI of the difference. *p < 0.05,^#^ *p* < 0.10; adjusted by age, sex, and BMI.

Overall, BMI *z*-score did not change (Pre: 0.52 ± 1.31, Post: 0.51 ± 1.30; mean difference: −0.01; 95%CI: −0.03 to 0.01; *p* = 0.192; Figure 1A) and age did not influence the results (Figure 1B). Females tended to increase BMI *z*-score whereas males reduced it (Figure 1C). Underweight and lean participants showed increased BMI *z*-score; in contrast, participants with overweight and obesity experienced reductions in BMI *z*-score (Pre: 1.45 ± 0.30, Post: 1.38 ± 0.51; difference: −0.08; 95%CI: −0.11 to −0.05; *p* < 0.001; and Pre: 2.65 ± 0.51, Post: 2.50 ± 0.60; difference: −0.15; 95%CI: −0.19 to −0.11; *p* < 0.001; Figure 1D).

## Discussion

This intensive, intersectoral intervention did not change the prevalence of physically inactive or sedentary youth at a city level. However, the intervention improved PA levels in physically inactive participants and mitigated BMI *z*-score among participants with overweight and obesity.

Young people show a consistent desire to be active, but are often constrained by environmental factors, such as school policy or curricula and physical structure (3). However, to increase external validity, this intervention was primarily focused on education-based activities, since decision makers could consider robust changes in physical and social environment as not feasible, due to budget restriction and/or inflexible education-related laws. The current data, nonetheless, suggest that community-based interventions with a stronger educational component may be not sufficient to elicit major changes in PA and sedentary behavior at a city level if not supplemented with more pronounced environmental changes, such as increasing the number of physical education classes and promoting extra class activities (e.g., organized sports) (14).

Although the intervention was not able to increase the proportion of physically active individuals, adolescents, and physically inactive individuals increased PA levels (3.3 and 11.2 min/day). Despite the relatively low magnitude of changes (especially in the former), one may argue that these increases may be relevant in real-life, considering that even small increases in PA may result in benefits, especially for physically inactive individuals (10), who were the vast majority in this study (70.6%).

The proportion of sedentary lifestyle among youth was dramatically high in this study (76.8%), supporting previous Latin-American surveys (15, 16). Importantly, the intervention decreased time spent watching television (during weekdays) and playing videogames, whereas time spent on the computer was increased. It appears that, in a real-world scenario, different types of sedentary activities may be differentially affected by a lifestyle intervention, and possibly replaced one by another over the course of time through unknown circumstances. There has been a worldwide reduction in time spent watching television, in parallel to an increase in time spent on the computer, a pattern that has been observed in Brazil (15) and in other developing countries (17). This trend needs to be considered by policymakers while elaborating programs aimed at limiting the use of different screen-based entertainments.

The reduction in BMI *z*-score experienced by participants with overweight and obesity enhances the external validity of efficacy studies (18, 19). The lack of changes in PA or sedentary behavior allows speculating that BMI *z*-score improvements may be mainly attributed to changes in eating habits. Considering that parents and children mutually influence each other's behavior (20), one could speculate that the profound improvements in eating habits experienced by adults (unpublished data) could have also affected their children's habits, which was unfortunately not assessed.

The strengths of this study are (i) the real-world, city-wide nature of the intervention; (ii) the large sample enrolled (~43% of total school-aged population) with a relatively low attrition rate (~10.5%); and (iii) the intervention's dual-focus on tackling PA and sedentary behavior. However, this study has limitations. First, internal validity of this study may have been affected by the lack of a control city, which was not possible since São Paulo cities are highly heterogeneous regarding administration, public health policies, and demographic characteristics. Second, subjective measures of PA and sedentary behavior may have led to inaccuracies inherent to this sort of method. Third, it is possible that the intervention was too short to change behavior among children. Finally, heterogeneity across cities may demand further assessments of cross-community transferability and adaptability before implementation of policies.

This intervention was not able to change the proportion of physical inactivity and sedentary behavior in youth at a city level. Nonetheless, physically inactive individuals increased PA levels and participants with overweight and obesity experienced a reduction in BMI *z*-score, evidencing the relevance of the intervention. Education-based lifestyle programs should be supplemented with environmental changes to better tackle inactivity/sedentary behavior in the real-world.

## Ethics statement

This study was conducted in accordance with The Declaration of Helsinki and was approved by the local research ethics committee (Ethics Committee of the Centro Universitário de Jaguariúna approval #54320616.6.0000.5409).

## Author contributions

MA, AP, FB, and BG participated in the conception and design of the study. MA, AP, FB, GM, EB, and BG were involved in the acquisition of study data, doing statistical analyses, and supervising and training teams to disseminate the intervention and gather data. AP, FB, and BG drafted the manuscript with critical revision from MA, GM, and EB. MA obtained funding. All authors have seen and approved the final version of the manuscript for publication.

### Conflict of interest statement

The authors declare that the research was conducted in the absence of any commercial or financial relationships that could be construed as a potential conflict of interest.
