# Supplementary material for: Tackling Youth Inactivity and Sedentary Behavior in an Entire Latin America City
Source: Front Pediatr. 2018 Oct 11;6:298. doi: 10.3389/fped.2018.00298 (PMC6194316; doi:10.3389/fped.2018.00298)
Supplement: Supplementary file 1 [file Table_1.DOC]

**SUPPLEMENTAL MATERIAL**

**Tackling youth inactivity and sedentary behavior in an entire Latin America city**

Marcio Atalla^1^, BSc; Ana Jessica Pinto^2^, BSc; Gregore Iven Mielke^3,4^, PhD; Erica Passos Baciuk^5^, PhD; Fabiana Braga Benatti^2*^, PhD; Bruno Gualano^2*^, PhD

**Corresponding author:** Prof. Bruno Gualano

Applied Physiology & Nutrition Research Group. Rheumatology Division, School of Medicine FMUSP, University of Sao Paulo, Sao Paulo, SP, BR. Dr. Arnaldo Avenue, 455, 3rd floor, ZIP code: 01246-903, Sao Paulo/SP, Brazil. Phone: +55 11 3061-8789; Fax: +55 11 3061-7490; e-mail: [gualano@usp.br](mailto:gualano@usp.br)

Summary

[File S1. Basis, aims and activities/policies of the “Life of Heath” intervention, according to the Behavior Change Wheel.^2^ 2](#__RefHeading___Toc517977186)

[Figure S1. Participants flow. 4](#__RefHeading___Toc517977187)

[Figure S2. Effects of the intervention (overall and stratified by age, sex, and BMI) on the achievement of physical activity recommendation. Data are expressed as relative risk (95% CI). 5](#__RefHeading___Toc517977188)

[Figure S3. Effects of the intervention (overall and stratified by age, sex, and BMI) on excessive sedentary behavior. Data are expressed as relative risk (95% CI). 6](#__RefHeading___Toc517977189)

[Table S1. Demographic characteristics of Jaguariuna. 7](#__RefHeading___Toc517977190)

[Table S2. Baseline characteristics of children and adolescents (n = 3,592). 8](#__RefHeading___Toc517977191)

[Table S3. Physical activity, sedentary behavior, and BMI z-score, before and after the intervention in children (n = 720). 9](#__RefHeading___Toc517977192)

[Table S4. Physical activity, sedentary behavior, and BMI z-score, before and after the intervention in adolescents (n = 1316). 10](#__RefHeading___Toc517977193)

[Table S5. Physical activity, sedentary behavior, and BMI z-score, before and after the intervention in females (n = 1120). 11](#__RefHeading___Toc517977194)

[Table S6. Physical activity, sedentary behavior, and BMI z-score, before and after the intervention in males (n = 916). 12](#__RefHeading___Toc517977195)

[Table S7. Physical activity, sedentary behavior, and BMI z-score, before and after the intervention in underweight participants (n = 47). 13](#__RefHeading___Toc517977196)

[Table S8. Physical activity, sedentary behavior, and BMI z-score, before and after the intervention in lean participants (n = 1324). 14](#__RefHeading___Toc517977197)

[Table S9. Physical activity, sedentary behavior, and BMI z-score, before and after the intervention in overweight participants (n = 420). 15](#__RefHeading___Toc517977198)

[Table S10. Physical activity, sedentary behavior, and BMI z-score, before and after the intervention in obese participants (n = 263). 16](#__RefHeading___Toc517977199)

[Table S11. Physical activity, sedentary behavior, and BMI z-score, before and after the intervention in inactive participants (n = 1593). 17](#__RefHeading___Toc517977200)

[Table S12. Physical activity, sedentary behavior, and BMI z-score, before and after the intervention in sedentary participants (n = 1563). 18](#__RefHeading___Toc517977201)

# **File S1. Basis, aims and activities/policies of the “Life of Heath” intervention, according to the Behavior Change Wheel.^2^**


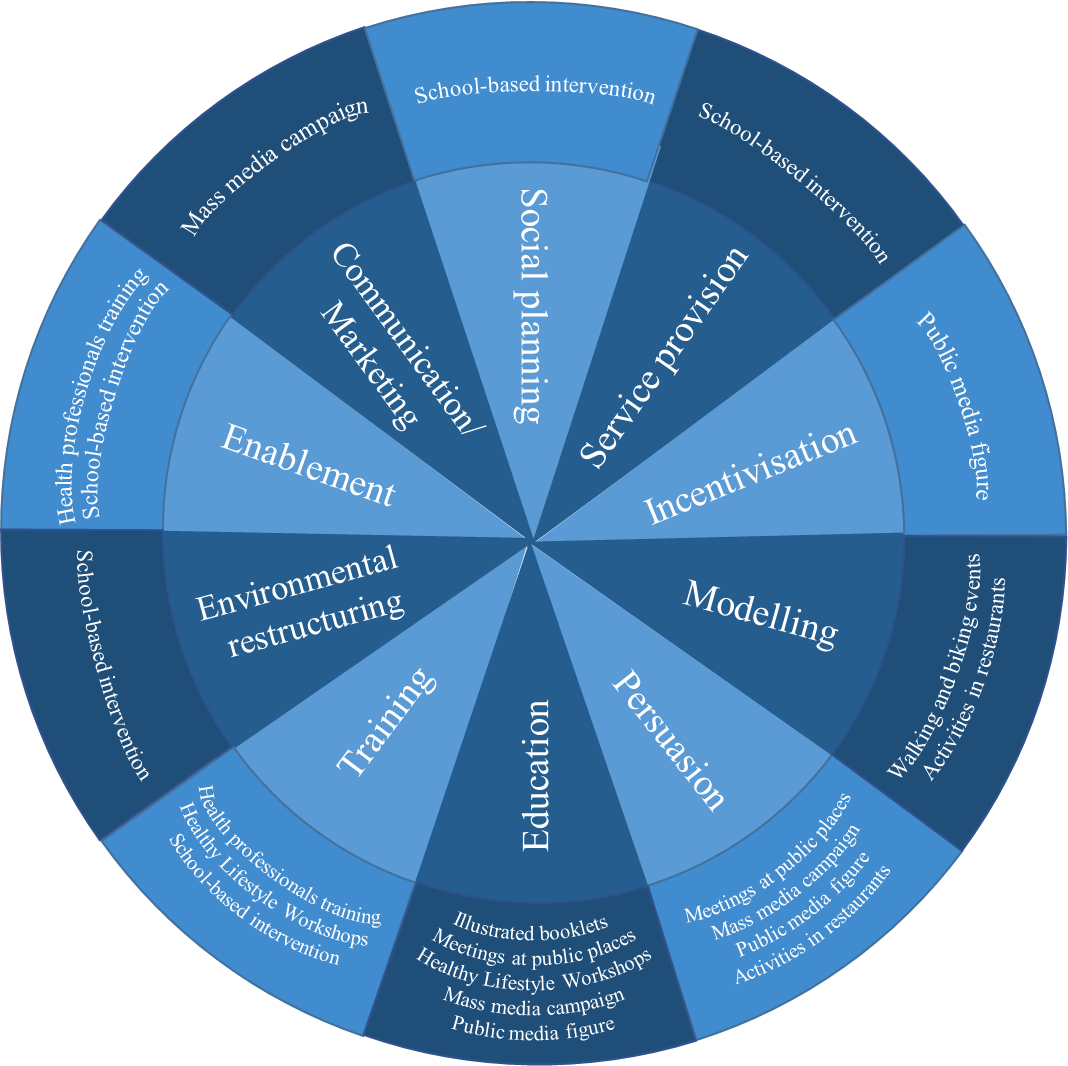


**Aims of the intervention:**

The “Life of Health” was a 7-month city-wide intervention program aimed at increasing physical activity levels and improving eating habits of the citizens of Jaguariuna, Brazil.

**Basis of the intervention:**

The promotion of physical activity focused on three pillars: 1) decreasing prolonged sedentary time (e.g., breaking prolonged hours spent sitting by taking hourly walks at work or home, or standing); 2) increasing structured physical activity (e.g., attending exercise classes at local gyms and parks, going on regular walks or jogging, etc.); and 3) increasing non-structured physical activity (e.g., increasing physical activity during leisure, walking or biking to work, taking the stairs instead of the elevator, etc.). Based on this, we created an illustrated booklet with 5 steps to increase physical activity which were: 1) Move throughout the day – take the stairs and walk more during the day; 2) Include some structured physical activity in your routine – walking 30 min a day, 5 days per week is enough; 3) If possible, do some sort of resistance exercise, it helps to preserve your muscles and bones; 4) It is never too soon or too late – kids and the elderly should also exercise regularly; and 5) Stand up – stand whenever possible while talking on the phone, watching TV, talking to friends, etc. The promotion of healthy eating habits was based on the Dietary Guideline for the Brazilian Population, released by the Ministry of Health in 2015.^3^ This is a novel guideline in which foods are no longer classified by their nutrient composition, but rather, by their level of processing. The main focus of the guideline is to promote the intake of fresh foods, i.e., natural and minimally processed foods (e.g., fresh fruits, legumes, roots, vegetables, meat, fresh milk, grains, etc.), making them the foundation of the diet, and to discourage the intake of commercially processed foods, i.e., foods to which several artificial additives and preservatives are added (e.g., salty fatty packaged snacks, cookies, sweetened juices, soft drinks, sweetened breakfast cereals, instant noodles, cake mix, hot dogs, poultry and fish nuggets, flavored yogurts and dairy drinks, etc.). Moreover, the guideline also promotes a healthier relationship and attitude to food by stimulating cooking and eating fresh foods in appropriate places along with family members whenever possible, making time to eat with full attention, and reconnecting with signs of hunger and satiety.

**Activities/interventions carried out to achieve healthy lifestyle:**

**Health professionals training:** Nurses, dietitians, doctors and health agents of public health centers and hospitals were educated about the importance of physical activity and healthy eating habits and how to instruct and motivate the population to improve them using a social constructional perspective.

**Illustrated booklets with 5 steps towards healthy eating and increasing physical activity:** Messages contained in the booklets (e.g.: “Move throughout the day - take the stairs and more walks during the day) were displayed on health centers, hospitals and companies.

**Walking and biking events:** Two walks and one biking event were promoted at the beginning and midway through the intervention.

**Meetings at public places:** A public media figure along with professional staff visited parks and public health centers and talked to the population about ways to improving eating and physical activity habits.

**Healthy Eating Workshop:** This workshop took place in a public park and was composed of nine stations.

**Exercise workshop:** This workshop was conducted in a public park where examples (in practice) on how exercise can be beneficial to metabolism and examples of exercises which can be done at home or included in routine were shown.

**Mass media campaign:** Information regarding ways to increase physical activity levels and improve eating habits was advertised via billboards and posters across the city, weekly text messages (SMS), weekly videos on a YouTube channel, weekly texts on a Facebook page and a website, and on a weekly show on a national level radio station.

**Activities in restaurants:** Restaurants were encouraged to offer a “healthy option” comprising a balanced meal composed of fresh foods only.

**Public media figure:** A public media figure was present at every intervention of the study to intensify compliance to the activities, give prizes to the participants, as well as give lectures at parks, companies and schools.

**School-based intervention:** Teachers, pedagogical and education coordinators and directors, along with the Secretary of Education of the city, were trained to disseminate a 5-goal program focused on increasing physical activity levels and reducing sedentary behavior, as thoroughly described in the manuscript.

# **Figure S1. Participants flow.**


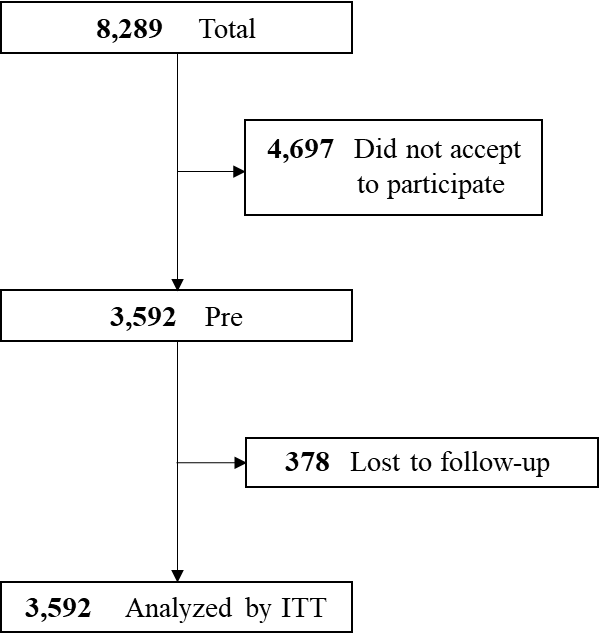


# **Figure S2. Effects of the intervention (overall and stratified by age, sex, and BMI) on the achievement of physical activity recommendation. Data are expressed as relative risk (95% CI).**


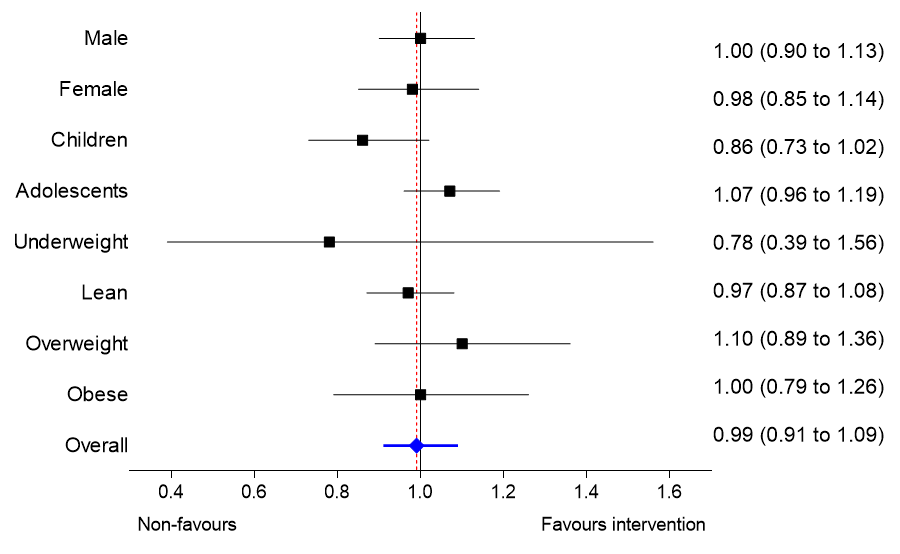


# **Figure S3. Effects of the intervention (overall and stratified by age, sex, and BMI) on excessive sedentary behavior. Data are expressed as relative risk (95% CI).**


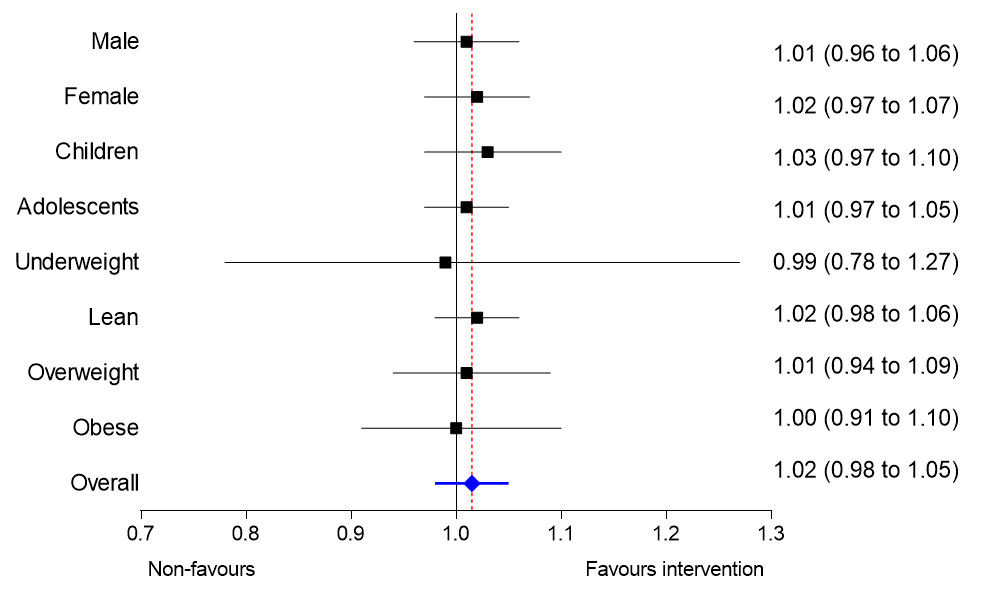


# **Table S1. Demographic characteristics of Jaguariuna.**

| **Characteristic*** |  |
| --- | --- |
| City | Jaguariuna |
| State | São Paulo |
| Region | Southeast Brazil |
| Country | Brazil |
| Area, km^2^ | 141·39 |
| Human Development Index | 0·78 |
| Gross Domestic Product per capita, USD | 42,471·63 |
| Population density, inhabitants/km^2^ | 313·37 |
| Estimated population in 2016, inhabitants | 53,069 |
| Population, inhabitants | 44,311 |
| Urban | 43,033 |
| Rural | 1,278 |
| Males | 22,004 |
| Females | 22,307 |
| 0 – 9 years | 5,888 |
| 10 – 19 years | 7,071 |
| 20 – 59 years | 26,635 |
| ≥ 60 years | 4,717 |
| Children and adolescents at school age (6 to 17 years), N | 8,289 |
| Schools, N | 18 |
| Life expectancy, years | 75·4 |
| Literacy rate, % | 92·5 |
| Literacy rate (6 to 14 years), % | 98·0 |
| Unemployment rate, % | 25·8 |

^*^Data from IBGE (Brazilian Institute of Geography and Statistics; <http://cod.ibge.gov.br/FHU>) and Jaguariuna City Council (<http://www.jaguariuna.sp.gov.br/>).

**Table S2. Baseline characteristics of children and adolescents (n = 3,592).**

| **Characteristic** | **Mean ± SD or n (%)** | **95% CI** | |
| --- | --- | --- | --- |
|  |  | **Lower** | **Upper** |
| Age, years | 10.8 ± 3.2 | 10.7 | 10.9 |
| Sex, N (%) |  |  |  |
| Female | 1978 (55.1%) | - | - |
| Male | 1614 (44.9%) | - | - |
| Weight, kg | 43.1 ± 17.3 | 42.6 | 43.7 |
| Height, cm | 145.9 ± 17.4 | 145.3 | 146.5 |
| Body mass index z-score | 0.52 ± 1.31 | 0.48 | 0.56 |
| Body mass index, N (%) |  |  |  |
| Underweight | 75 (2.1%) | - | - |
| Lean | 2255 (62.8%) |  |  |
| Overweight | 733 (20.4%) | - | - |
| Obese | 529 (14.7%) | - | - |

# **Table S3. Physical activity, sedentary behavior, and BMI z-score, before and after the intervention in children (n = 720).**

| **Variable** | **Pre** | **Post** | **Delta change** | **95% CI** | **p** |
| --- | --- | --- | --- | --- | --- |
| Physical activity, min/day | 39·8 ± 54·6 | 34·6 ± 53·7 | -5·2 | -9·9 to -0·4 | 0·033 |
| Physically active, N (%) | 151 (21·0%) | 130 (17·8%) | 0·86^*^ | 0·73 to 1·02^*^ | 0·096 |
| Television time during weekdays, min/day | 136·3 ± 101·2 | 126·0 ± 97·1 | -11·4 | -18·1 to -4·6 | 0·001 |
| Television time during weekend days, min/day | 67·6 ± 68·4 | 71·3 ± 76·8 | 3·8 | -1·4 to 9·1 | 0·152 |
| Time spent playing video games, min/day | 58·8 ± 93·0 | 41·7 ± 77·7 | -18·6 | -25·1 to -12·0 | <0·001 |
| Time spent on computer, min/day | 47·6 ± 88·7 | 85·3 ± 107·0 | 38·7 | 31·5 to 45·9 | <0·001 |
| Sedentary (> 2h/day), N (%) | 512 (71·1%) | 536 (73·2%) | 1·03^*^ | 0·97 to 1·10^*^ | 0·263 |
| Body mass index z-score | 0·59 ± 1·34 | 0·59 ± 1·33 | -0·01 | -0·04 to 0·01 | 0·232 |

Data are expressed as mean ± standard deviation, delta change (Post - Pre), and 95% CI of the difference unless stated otherwise. ^*^Relative risk. P value was adjusted by age, sex, and BMI.

# **Table S4. Physical activity, sedentary behavior, and BMI z-score, before and after the intervention in adolescents (n = 1316).**

| **Variable** | **Pre** | **Post** | **Delta change** | **95% CI** | **p** |
| --- | --- | --- | --- | --- | --- |
| Physical activity, min/day | 35·8 ± 50·8 | 39·2 ± 57·2 | 3·3 | -0·1 to 6·7 | 0·057 |
| Physically active, N (%) | 292 (22·2%) | 269 (23·2%) | 1·07^*^ | 0·96 to 1·19^*^ | 0·679 |
| Television time during weekdays, min/day | 124·8 ± 107·2 | 119·0 ± 109·9 | -7·0 | -12·4 to -1·6 | 0·011 |
| Television time during weekend days, min/day | 86·1 ± 84·2 | 82·6 ± 87·7 | -1·9 | -7·0 to 3·2 | 0·470 |
| Time spent playing video games, min/day | 56·6 ± 105·5 | 35·3 ± 83·1 | -22·5 | -27·8 to -17·2 | <0·001 |
| Time spent on computer, min/day | 118·2 ± 155·7 | 189·2 ± 198·7 | 82·2 | 71·4 to 93·0 | <0·001 |
| Sedentary (> 2h/day), N (%) | 1051 (79·8%) | 938 (80·9%) | 1·01^*^ | 0·97 to 1·05^*^ | 0·132 |
| Body mass index z-score | 0·40 ± 1·26 | 0·38 ± 1·26 | -0·01 | -0·04 to 0·02 | 0·521 |

Data are expressed as mean ± standard deviation, delta change (Post - Pre), and 95% CI of the difference unless stated otherwise. ^*^Relative risk. P value was adjusted by age, sex, and BMI.

# **Table S5. Physical activity, sedentary behavior, and BMI z-score, before and after the intervention in females (n = 1120).**

| **Variable** | **Pre** | **Post** | **Delta change** | **95% CI** | **p** |
| --- | --- | --- | --- | --- | --- |
| Physical activity, min/day | 28·2 ± 46·1 | 27·7 ± 47·1 | -1·2 | -4·4 to 2·1 | 0·482 |
| Physically active, N (%) | 174 (15·5%) | 158 (15·2%) | 0·98^*^ | 0·85 to 1·14^*^ | 0·528 |
| Television time during weekdays, min/day | 137·3 ± 109·6 | 126·7 ± 107·0 | -12·4 | -18·2 to -6·7 | <0·001 |
| Television time during weekend days, min/day | 81·4 ± 81·9 | 80·7 ± 85·6 | 1·0 | -3·9 to 5·8 | 0·696 |
| Time spent playing video games, min/day | 49·1 ± 101·8 | 28·3 ± 78·2 | -22·5 | -28·2 to -16·8 | <0·001 |
| Time spent on computer, min/day | 98·1 ± 152·6 | 153·5 ± 185·5 | 64·6 | 54·3 to 74·8 | <0·001 |
| Sedentary (> 2h/day), N (%) | 848 (75·7%) | 800 (77·1%) | 1·02^*^ | 0·97 to 1·07^*^ | 0·173 |
| Body mass index z-score | 0·47 ± 1·27 | 0·50 ± 1·26 | 0·02 | 0·00 to 0·05 | 0·052 |

Data are expressed as mean ± standard deviation, delta change (Post - Pre), and 95% CI of the difference unless stated otherwise. ^*^Relative risk. P value was adjusted by age, sex, and BMI.

# **Table S6. Physical activity, sedentary behavior, and BMI z-score, before and after the intervention in males (n = 916).**

| **Variable** | **Pre** | **Post** | **Delta change** | **95% CI** | **p** |
| --- | --- | --- | --- | --- | --- |
| Physical activity, min/day | 48·2 ± 57·0 | 49·3 ± 63·1 | 1·2 | -3·4 to 5·9 | 0·602 |
| Physically active, N (%) | 269 (29·4%) | 241 (28·3%) | 1·00^*^ | 0·90 to 1·13^*^ | 0·544 |
| Television time during weekdays, min/day | 118·6 ± 98·7 | 115·6 ± 102·6 | -3·7 | -9·8 to 2·3 | 0·228 |
| Television time during weekend days, min/day | 77·4 ± 76·3 | 75·2 ± 81·5 | -1·0 | -6·8 to 4·9 | 0·744 |
| Time spent playing video games, min/day | 67·5 ± 99·8 | 49·2 ± 83·0 | -19·2 | -25·2 to -13·2 | <0·001 |
| Time spent on computer, min/day | 87·3 ± 122·5 | 143·5 ± 165·0 | 62·9 | 52·7 to 73·1 | <0·001 |
| Sedentary (> 2h/day), N (%) | 715 (78·1%) | 674 (79·0%) | 1·01^*^ | 0·96 to 1·06^*^ | 0·331 |
| Body mass index z-score | 0·58 ± 1·36 | 0·51 ± 1·36 | -0·05 | -0·07 to -0·03 | <0·001 |

Data are expressed as mean ± standard deviation, delta change (Post - Pre), and 95% CI of the difference unless stated otherwise. ^*^Relative risk. P value was adjusted by age, sex, and BMI.

# **Table S7. Physical activity, sedentary behavior, and BMI z-score, before and after the intervention in underweight participants (n = 47).**

| **Variable** | **Pre** | **Post** | **Delta change** | **95% CI** | **p** |
| --- | --- | --- | --- | --- | --- |
| Physical activity, min/day | 30·5 ± 47·6 | 38·5 ± 58·5 | -5·7 | -17·9 to 6·6 | 0·364 |
| Physically active, N (%) | 10 (21·3%) | 7 (17·5%) | 0·78^*^ | 0·39 to 1·56^*^ | 0·388 |
| Television time during weekdays, min/day | 136·2 ± 79·6 | 136·6 ± 85·1 | -3·1 | -28·6 to 22·4 | 0·814 |
| Television time during weekend days, min/day | 82·1 ± 65·8 | 81·5 ± 78·4 | -3·9 | -25·8 to -18·0 | 0·728 |
| Time spent playing video games, min/day | 56·8 ± 98·7 | 47·3 ± 85·3 | -40·4 | -75·2 to -5·5 | 0·023 |
| Time spent on computer, min/day | 90·0 ± 158·7 | 124·4 ± 165·4 | 57·3 | 18·5 to 96·2 | 0·004 |
| Sedentary (> 2h/day), N (%) | 35 (74·5%) | 31 (73·8%) | 0·99^*^ | 0·78 to 1·27^*^ | 0·621 |
| Body mass index z-score | -2·42 ± 0·39 | -2·11 ± 0·84 | 0·30 | 0·10 to 0·49 | 0·001 |

Data are expressed as mean ± standard deviation, delta change (Post - Pre), and 95% CI of the difference unless stated **otherwise. ^*^Relative risk. P value was adjusted by age, sex, and BMI.**

# **Table S8. Physical activity, sedentary behavior, and BMI z-score, before and after the intervention in lean participants (n = 1324).**

| **Variable** | **Pre** | **Post** | **Delta change** | **95% CI** | **p** |
| --- | --- | --- | --- | --- | --- |
| Physical activity, min/day | 38·0 ± 53·2 | 37·7 ± 53·3 | -1·2 | -4·6 to 2·3 | 0·499 |
| Physically active, N (%) | 295 (22·3%) | 256 (23·2%) | 0·97^*^ | 0·87 to 1·08^*^ | 0·262 |
| Television time during weekdays, min/day | 129·3 ± 105·7 | 123·2 ± 107·3 | -6·7 | -11·9 to -1·4 | 0·013 |
| Television time during weekend days, min/day | 80·5 ± 81·3 | 80·8 ± 84·8 | 0·5 | -4·3 to 5·2 | 0·844 |
| Time spent playing video games, min/day | 52·7 ± 95·3 | 41·5 ± 85·6 | -17·5 | -22·5 to -12·6 | <0·001 |
| Time spent on computer, min/day | 104·2 ± 151·0 | 143·2 ± 176·3 | 68·4 | 59·1 to 77·7 | <0·001 |
| Sedentary (> 2h/day), N (%) | 1021 (77·1%) | 956 (78·7%) | 1·02^*^ | 0·98 to 1·06^*^ | 0·097 |
| Body mass index z-score | -0·18 ± 0·73 | -0·15 ± 0·83 | 0·03 | 0·01 to 0·05 | 0·009 |

Data are expressed as mean ± standard deviation, delta change (Post - Pre), and 95% CI of the difference unless stated otherwise. ^*^Relative risk. P value was adjusted by age, sex, and BMI.

# **Table S9. Physical activity, sedentary behavior, and BMI z-score, before and after the intervention in overweight participants (n = 420).**

| **Variable** | **Pre** | **Post** | **Delta change** | **95% CI** | **p** |
| --- | --- | --- | --- | --- | --- |
| Physical activity, min/day | 37·1 ± 54·2 | 39·9 ± 62·6 | 4·4 | -2·3 to 11·1 | 0·197 |
| Physically active, N (%) | 77 (19·2%) | 79 (22·6%) | 1·10^*^ | 0·89 to 1·36^*^ | 0·413 |
| Television time during weekdays, min/day | 124·6 ± 99·4 | 122·2 ± 102·3 | -11·1 | -20·6 to -1·5 | 0·024 |
| Television time during weekend days, min/day | 78·5 ± 84·2 | 76·5 ± 85·5 | 0·3 | -8·3 to 8·9 | 0·942 |
| Time spent playing video games, min/day | 52·8 ± 95·6 | 34·7 ± 78·5 | -24·6 | -33·4 to -15·9 | <0·001 |
| Time spent on computer, min/day | 105·0 ± 148·6 | 135·8 ± 169·9 | 59·6 | 43·2 to 76·0 | <0·001 |
| Sedentary (> 2h/day), N (%) | 306 (76·1%) | 292 (77·0%) | 1·01^*^ | 0·94 to 1·09^*^ | 0·652 |
| Body mass index z-score | 1·45 ± 0·30 | 1·38 ± 0·51 | -0·08 | -0·11 to -0·05 | <0·001 |

Data are expressed as mean ± standard deviation, delta change (Post - Pre), and 95% CI of the difference unless stated otherwise. ^*^Relative risk. P value was adjusted by age, sex, and BMI.

# **Table S10. Physical activity, sedentary behavior, and BMI z-score, before and after the intervention in obese participants (n = 263).**

| **Variable** | **Pre** | **Post** | **Delta change** | **95% CI** | **p** |
| --- | --- | --- | --- | --- | --- |
| Physical activity, min/day | 32·2 ± 44·0 | 34·4 ± 51·7 | 0·4 | -6·6 to 7·3 | 0·920 |
| Physically active, N (%) | 61 (23·2%) | 57 (25·3%) | 1·00^*^ | 0·79 to 1·26^*^ | 0·693 |
| Television time during weekdays, min/day | 123·5 ± 110·6 | 116·8 ± 106·9 | -15·2 | -26·3 to -4·0 | 0·008 |
| Television time during weekend days, min/day | 74·7 ± 74·9 | 78·0 ± 79·8 | -1·8 | -11·2 to 7·5 | 0·700 |
| Time spent playing video games, min/day | 66·4 ± 115·1 | 46·7 ± 97·7 | -28·6 | -41·9 to -15·3 | <0·001 |
| Time spent on computer, min/day | 94·7 ± 134·3 | 122·2 ± 151·8 | 47·8 | 30·4 to 65·1 | <0·001 |
| Sedentary (> 2h/day), N (%) | 201 (76·4%) | 195 (76·5%) | 1·00^*^ | 0·91 to 1·10^*^ | 0·771 |
| Body mass index z-score | 2·65 ± 0·51 | 2·50 ± 0·60 | -0·15 | -0·19 to -0·11 | <0·001 |

Data are expressed as mean ± standard deviation, delta change (Post - Pre), and 95% CI of the difference unless stated otherwise. ^*^Relative risk. P value was adjusted by age, sex, and BMI.

# **Table S11. Physical activity, sedentary behavior, and BMI z-score, before and after the intervention in inactive participants (n = 1593).**

| **Variable** | **Pre** | **Post** | **Delta change** | **95% CI** | **p** |
| --- | --- | --- | --- | --- | --- |
| Physical activity, min/day | 15·8 ± 17·0 | 26·4 ± 43·4 | 11·2 | 8·8 to 13·6 | <0·001 |
| Television time during weekdays, min/day | 130·5 ± 105·7 | 122·5 ± 104·6 | -9·5 | -14·4 to -4·7 | <0·001 |
| Television time during weekend days, min/day | 79·9 ± 79·9 | 78·9 ± 84·2 | 0·3 | -3·8 to 4·4 | 0·874 |
| Time spent playing video games, min/day | 55·0 ± 102·2 | 35·7 ± 81·1 | -19·8 | -24·4 to -15·2 | <0·001 |
| Time spent on computer, min/day | 99·1 ± 144·9 | 150·8 ± 178·1 | 62·5 | 54·2 to 70·8 | <0·001 |
| Body mass index z-score | 0·44 ± 1·30 | 0·42 ± 1·29 | -0·01 | -0·04 to 0·01 | 0·304 |

Data are expressed as mean ± standard deviation, delta change (Post - Pre), and 95% CI of the difference unless stated otherwise. P value was adjusted by age, sex, and BMI.

# **Table S12. Physical activity, sedentary behavior, and BMI z-score, before and after the intervention in sedentary participants (n = 1563).**

| **Variable** | **Pre** | **Post** | **Delta change** | **95% CI** | **p** |
| --- | --- | --- | --- | --- | --- |
| Physical activity, min/day | 37·3 ± 52·4 | 38·2 ± 56·6 | 0·3 | -2·8 to 3·4 | 0·866 |
| Television time during weekdays, min/day | 82·7 ± 103·4 | 87·4 ± 103·4 | -17·0 | -22·1 to -11·9 | <0·001 |
| Television time during weekend days, min/day | 94·4 ± 89·5 | 90·4± 91·5 | -2·6 | -7·2 to 2·0 | 0·268 |
| Time spent playing video games, min/day | 64·7 ± 95·3 | 49·6 ± 83·4 | -29·2 | -34·3 to -24·0 | <0·001 |
| Time spent on computer, min/day | 78·6 ± 127·6 | 119·0 ± 162·4 | 58·8 | 50·3 to 67·3 | <0·001 |
| Body mass index z-score | 0·52 ± 1·31 | 0·51 ± 1·30 | -0·03 | -0·05 to 0·00 | 0·024 |

Data are expressed as mean ± standard deviation, delta change (Post - Pre), and 95% CI of the difference unless stated otherwise. P value was adjusted by age, sex, and BMI.

**REFERENCES**

1. Des Jarlais DC, Lyles C, Crepaz N, Group T. Improving the reporting quality of nonrandomized evaluations of behavioral and public health interventions: the TREND statement. *Am J Public Health* 2004; **94**(3): 361-6.

2. Michie S, van Stralen MM, West R. The behaviour change wheel: a new method for characterising and designing behaviour change interventions. *Implement Sci* 2011; **6**: 42.

3. Ministry of Health of Brazil. Secretariat of Health Care. Primary Health Care Department. Dietary Guidelines for the Brazilian population / Ministry of Health of Brazil, Secretariat of Health Care, Primary Health Care Department ; translated by Carlos Augusto Monteiro. . Brasília: Ministry of Health of Brazil; 2015. p. 150 p. : il.
